# MetAMOS: a modular and open source metagenomic assembly and analysis pipeline

**DOI:** 10.1186/gb-2013-14-1-r2

**Published:** 2013-01-15

**Authors:** Todd J Treangen, Sergey Koren, Daniel D Sommer, Bo Liu, Irina Astrovskaya, Brian Ondov, Aaron E Darling, Adam M Phillippy, Mihai Pop

**Affiliations:** 1 Center for Bioinformatics and Computational Biology, 3125 Biomolecular Sciences Bldg #296, University of Maryland, College Park, MD 20742, USA; 2 National Biodefense Analysis and Countermeasures Center, 110 Thomas Johnson Drive, Frederick, MD 21702, USA; 3 Department of Computer Science, AV Williams Building, University of Maryland, College Park, MD 20742, USA; 4 Genome Center, 451 Health Sciences Drive, University of California, Davis, California 95616, USA

## Abstract

We describe MetAMOS, an open source and modular metagenomic assembly and analysis pipeline. MetAMOS represents an important step towards fully automated metagenomic analysis, starting with next-generation sequencing reads and producing genomic scaffolds, open-reading frames and taxonomic or functional annotations. MetAMOS can aid in reducing assembly errors, commonly encountered when assembling metagenomic samples, and improves taxonomic assignment accuracy while also reducing computational cost. MetAMOS can be downloaded from: https://github.com/treangen/MetAMOS.

## Rationale

Metagenomics has opened the door for unprecedented studies of microbial communities sampled from the environment (for example, ocean surveys [1-3], Antarctic expeditions [4], and even health-care facilities [5]), as well as from living organisms [6] and the human body [7-11]. These studies have been made possible by dramatic recent advances in high-throughput sequencing technologies, the same technologies that have revolutionized the study of individual genomes, such as recent efforts to reconstruct the genomes of thousands of humans [12]. While sequencing technologies have been rapidly improving, the computational infrastructure needed to analyze the resulting data has been slow to adapt to the volume and characteristics of the data being generated. In particular, genome assembly, though substantially improved in recent years [13], remains an important challenge even for single organisms. In metagenomic projects, traditional genome assemblers have trouble disentangling closely related strains and distinguishing true polymorphisms from sequencing errors. As a result, many researchers forgo assembly and instead focus their analyses directly on the underlying reads [14-22]. While these methods have shown promise, analysis tasks such as gene finding and taxonomic classification become much easier when applied to genomic contigs reconstructed through assembly. Accordingly, a number of computational tools specifically targeted at metagenomic *de novo *assembly have begun to emerge [23-26]. These tools are, however, still in their infancy and their application is limited by a number of factors such as: (i) performance issues when applied to large metagenomic datasets; (ii) the need for careful parameter tuning in order to optimize assembly results; and (iii) the lack of integration with the other components of metagenomic analysis pipelines. Furthermore, the relative benefits and drawbacks of individual assembly tools are difficult to ascertain given the lack of metagenomic reference datasets, and the widely divergent data characteristics of current metagenomic projects.

It is also important to stress that assembly is just one of many other bioinformatics analyses typically performed in metagenomic projects, including taxonomic classification, gene annotation, variant analysis, and so on. Performing these tasks requires the installation, integration, and tuning of multiple software packages, which is not trivial even for groups with extensive bioinformatics expertise. As a result, most studies rely on *ad hoc *pipelines based on custom scripts and intensive manual analyses, making it difficult to reproduce or extend analysis results and hampering collaboration.

To address these challenges, we developed MetAMOS, a modular and customizable framework for metagenomic assembly and analysis. To researchers without bioinformatics expertise, MetAMOS provides a push-button solution for analysis of metagenomic datasets, irrespective of the sequencing technology used. In addition to the actual assembly, MetAMOS outputs a taxonomic profile of the community, gene predictions, and potential genomic variants. In some sense, MetAMOS can be viewed as an assembly-centric counterpart to QIIME [27] and mothur [28], popular pipelines used for the analysis 16S rRNA data. To bioinformaticians, MetAMOS provides a modular and flexible pipeline, integrating many metagenomic analysis tools that can be tailored and extended to meet specific analysis needs.

## Overview of the MetAMOS analysis pipeline

**Figure 1:**
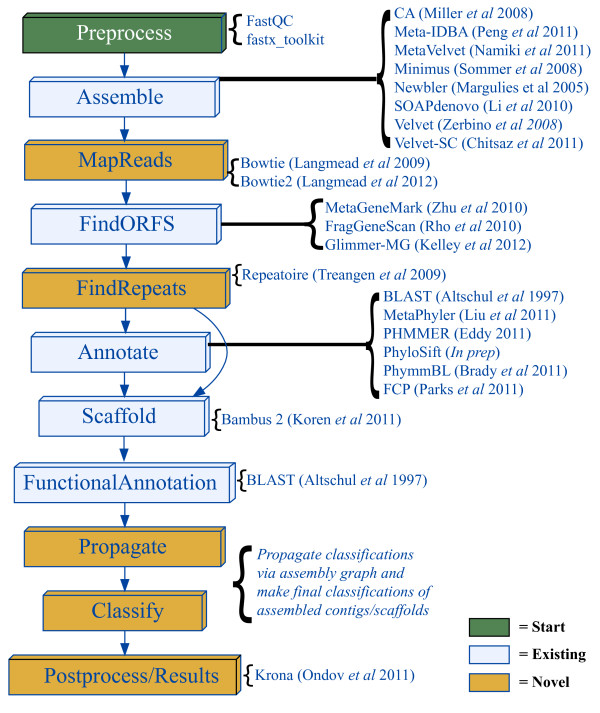
**MetAMOS workflow**. The arrows indicate dependence between pipeline steps. MetAMOS leverages over 20 existing analysis tools for various steps in the pipeline. The figure also highlights the novel contributions to metagenomic analysis made by MetAMOS. Miller *et al*. 2008 [33]; Peng *et al*. 2011 [25]; Namiki *et al*. 2011 [24]; Sommer *et al*. 2008 [40]; Margulies *et al*. 2005 [99]; Li *et al*. 2010 [51]; Zerbino *et al*. 2008 [63]; Chitsaz *et al*. 2011 [64]; Langmead *et al*. 2010 [49]; Langmead *et al*. 2012 [50]; Zhu *et al*. 2010 [100]; Rho *et al*. 2010 [68]; Kelley *et al*. 2012 [69]; Treangen *et al*. 2009 [75]; Altschul *et al*. 1990 [66]; Liu *et al*. 2011 [52]; Eddy *et al*. 2011 [67]; Brady *et al*. 2011 [14]; Parks *et al*. 2011 [22]; Koren *et al*. 2011 [30]; Ondov *et al*. 2011 [46].

The MetAMOS package is built around a collection of publicly available assembly and analysis tools tied together with the help of the lightweight workflow system Ruffus [29]. The current analysis workflow and available software packages are outlined in Figure 1, and discussed in detail below in the Workflow section. It is important to stress, however, that these tools are not simply strung together into *ad hoc *pipelines; rather, the entire pipeline is built around the unique features provided by the metagenomic scaffolder Bambus 2 [30].

**Figure 2:**
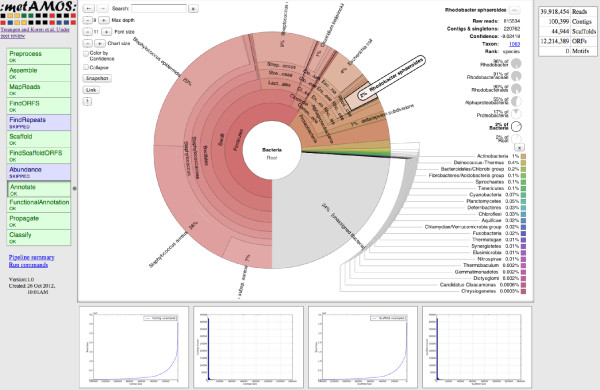
**MetAMOS HTML report interface**. The majority of results generated during the pipeline are exposed to the user via an interactive HTML report. From left to right. Pipeline status: this lists the state (OK, FAIL, SKIP) for each step in the pipeline. In addition, each individual step is linked to an image/text summary of the results generated during this step. Krona plot: by default, in the main section of the report an interactive Krona plot of the annotations is displayed. This main section is dynamically updated with content from the other steps at the user's request. Single/Multiple Sample plots: This section is dedicated to displaying automatically generated R plots of assembly statistics. If multiple samples are available, they can be combined into a single plot. Quick summary: this column, as the name implies, provides a very brief overview of the results (listing in descending order number of reads, contigs, scaffolds, ORFs, variants) in addition to linking to a file containing a summary of all of the executed commands for the MetAMOS run.

The pipeline can be broadly separated into three main sections. The first includes a pre-processing step aimed at constructing a collection of conservative contigs using software specific to the sequencing technology employed (Sanger, 454, and Illumina data are currently supported). Specifically, pre-processing involves the following steps: (1) dynamic library size re-estimation based on read mappings, and (2) contig cleaning (removal of contigs that lack read mappings). In the second step, Bambus 2 is used to identify genomic repeats, scaffold the initial set of contigs, correct assembly errors, extend contigs, and detect genomic variants. In a third, post-scaffolding stage, the contigs are further analyzed and annotated using scaffold-aware approaches, such as the propagation of taxonomic labels to all contigs linked together within a scaffold. Thus, the scaffold information generated by Bambus 2 allows us to integrate multiple sources of information and obtain more accurate annotations of the resulting assembly. At the end of the final stage, MetAMOS produces an interactive HTML report that summarizes the main results of the run (Figure 2).

## Related software

Our package shares similarities with SmashCommunity [31], a metagenomic analysis pipeline targeted at 454 and Sanger data. Unlike MetAMOS, SmashCommunity only supports a small set of assembly and analysis tools (Arachne [32], Celera Assembler [33,34], Forge, and MetaGeneMark [35]). More importantly, however, SmashCommunity simply links together the individual analysis tools and does not provide additional functionality made possible by the integration of different analyses. For these reasons, instead of building upon SmashCommunity, we decided to build MetAMOS around the AMOS open-source genome assembly framework, which already included many assembly-centric analysis utilities [30,36-41].

## Results

Below we demonstrate the use of MetAMOS and compare its performance to other software tools that can and have been used for metagenomic analysis. We focus our analysis on several datasets with complementary characteristics: 'mock' metagenomic communities from the Human Microbiome Project (HMP) [11], and real metagenomic samples from the HMP and the Metagenomics of the Human Intestinal Tract (MetaHIT) [42] projects. The mock communities (described in more detail below) comprise a known mixture of organisms and provide a valuable resource for assessing the accuracy of different assembly tools. The real datasets are a sample of data from recent studies and demonstrate the practical potential of our tool.

### HMP mock communities

#### Assembly analysis

Results obtained on real metagenomic samples are difficult to evaluate due to the absence of a 'golden truth' reference. Thus, to first compare and evaluate metagenomic assembly accuracy, we rely on metagenomic samples with known composition, specifically two 'mock' communities created by the HMP consortium [43,44]. These communities represent the result of sequencing a mixture of quantified DNA fragments from organisms with known genomic sequences, comprising over 50 bacterial genomes and a few eukaryotes. While not without limitations, this dataset has advantages over purely simulated data because it captures the error and bias introduced by the sequencing technology.

**Figure 3:**
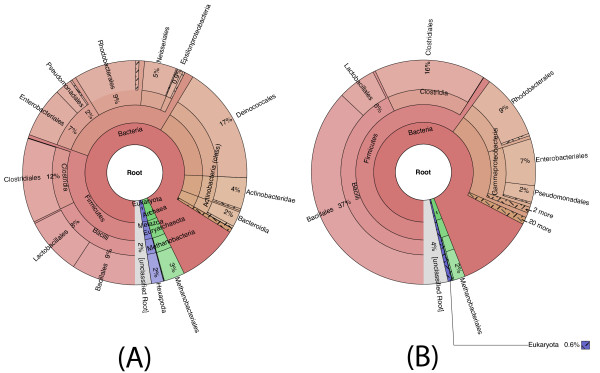
**Krona plots of the HMP mock Even and Staggered samples**. **(a) **HMP mock Even; **(b) **HMP mock Staggered. Meta-IDBA was used for assembly, MetaGeneMark for ORF prediction, and MetaPhyler for classification. The classifications can optionally be colored by average confidence, highlighting the certainty in the classifications.

Data from two HMP mock communities are available: Even and Staggered (NCBI BioProject ID 48475). The reference genomes in these mock communities are precisely known, the abundances are fairly well known, and the reads were sequenced with the Illumina GAII instrument [45]. We independently confirmed the different abundance profiles of the mock Even and Staggered communities with MetaPhyler; Figure 3 shows the interactive Krona [46] chart for these samples, as output by MetAMOS.

Using these datasets we evaluate the performance of eight different methods: SOAPdenovo (SOAPdenovo contigs), SOAPdenovo_MA (MetAMOS+SOAPdenovo unitigs), Meta-IDBA, Meta-IDBA_MA (MetAMOS+Meta-IDBA contigs), MetaVelvet, MetaVelvet_MA (MetAMOS+MetaVelvet unitigs), Velvet, Velvet_MA (MetAMOS+Velvet unitigs). The methods with the suffix '_MA' represent the use of the specific assembler within the MetAMOS framework, specifically to generate the initial high-confidence contigs that are then scaffolded and further analyzed with Bambus 2 and the other utilities provided by MetAMOS. Unitigs are sections of the genome that can be unambiguously reconstructed by an assembler on the basis of reads alone (entirely contained in either unique regions or repeats), that is, regions that do not span the boundary between repeats and unique regions.

**Figure 4:**
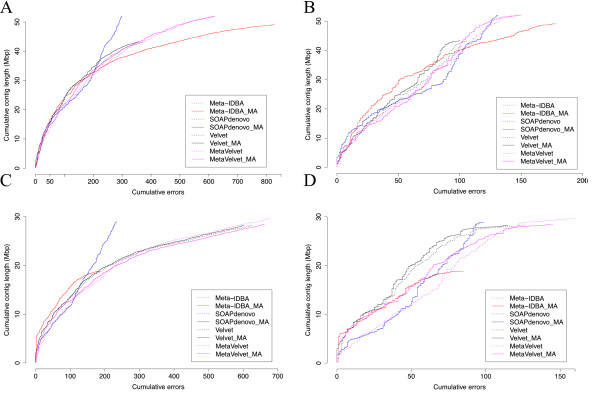
**Feature response curve performance on mock Even and mock Staggered communities**. The y-axis shows the cumulative contig length (sorted in decreasing order) and the x-axis shows the corresponding number of misassembled contigs. The thick lines (assemblies ending in _MA) represent the results obtained by running MetAMOS using the corresponding assembler (dashed lines) for the Assembly module. **(a) **HMP mock Even, all errors reported **(b) **HMP mock Even, heavy mis-assemblies only. **(c) **HMP mock Staggered, all errors reported. **(d) **HMP mock Staggered, heavy mis-assemblies only. For all but Velvet and MetaVelvet the dashed lines are hidden behind the solid lines because the original assemblies are mostly unchanged.

The results are shown in Figure 4a, b (mock Even), and Figure 4c, d (mock Staggered) using the recently proposed Feature Response Curve technique [47]. These curves simultaneously track the cumulative size of the assembly (total number of bases reconstructed) and the number of errors found in the assembly, and are similar in spirit to the well known receiver operating characteristic (ROC) used for comparing classifier systems. In a Feature Response Curve plot, the contigs are sorted by decreasing sequence length, and the number of errors and cumulative contig size are plotted along the x- and y-axes, respectively. When comparing two assemblies, A and B, if the curve corresponding to assembly A is above that of assembly B, one can infer that A contains more of the (meta)genome while incurring the same number of errors as assembly B, or stated differently, assembly A reconstructs the same amount of DNA as assembly B but with fewer errors.

An observation evident from the analysis of the 'mock Even' dataset (Figure 4a, b) is that metagenomic-specific assemblers (MetaVelvet and Meta-IDBA) have very similar performance to non-metagenomic assemblers. For example, Velvet appears to provide the best results within the most contiguous 37 Mbp of the assembly (roughly half of the total genomic content of the sample; Figure 4a). Beyond this point Velvet, and most of the other assemblers, rapidly accumulate errors while reconstructing the remaining genomic content of the sample. SOAPdenovo, the assembler used by both the HMP [43,44] and the MetaHIT project [48], has a more stable error characteristic, accumulating overall fewer errors than the other assemblers. However, SOAPdenovo also makes more mistakes within its larger contigs, as shown by the dip within the bottom left side of the curve. Because MetAMOS includes independent pre-processing and scaffolding routines, the performance of Velvet and MetaVelvet improved when run within the MetAMOS framework (Figure S1 in Additional file 1).

All assemblers have lower performance on the 'mock Staggered' community (Figure 4c, d), which is expected to better model the pattern of taxonomic diversity encountered in real data. Meta-IDBA shows strong early performance, but is only able to reconstruct approximately 25% of the reference genomes (20 out of 83 Mbp) in contigs larger than 150 bp. SOAPdenovo + MetAMOS obtains the best overall performance for this Staggered simulated dataset, and again, MetAMOS improves over the Velvet and MetaVelvet assemblers, with the gain being more pronounced than in the mock Even dataset.

Also evident from these figures is the inherent difficulty of metagenomic assembly. Even in the easiest community (mock Even), the best assembler can only reconstruct about 66% of the total genomic content (55 out of 83 Mbp) in contigs larger than 150 bp, while for the more complicated community, less than 30 Mbp are reconstructed (36%).

When analyzing mis-assemblies we observe that all assemblers make mistakes (Table 1), especially in the category termed 'heavy mis-assembly'. Heavy mis-assemblies are contigs with only one alignment to a reference genome covering less than 80% of the contig's length, or multiple incompatible alignments to a single reference. MetaVelvet has the best contiguity at 10 Mbp in both mock communities but also generates more assembly errors than the more conservative SOAPdenovo assembly. MetAMOS is able to improve upon MetaVelvet in terms of both contiguity and error rate in the mock Even dataset, while in the mock Staggered data the reduction in error is associated with a reduction in contiguity. In addition, MetAMOS nearly matches the stand-alone assemblers in terms of reference representation, while lowering the number of errors they produce (Table 1). This conservative approach is critical for ensuring the accuracy of downstream analyses.

**Table 1:** Comparison of assembly statistics

Dataset	Assembler	#ctgs/scfs	Good Ctgs/scfs	Total aln (Mbp)	Slt	Hvy	Ch	Size @ 10 Mbp	#@ 10 Mbp	Max ctg size	Err per Mbp
mockE	SOAPdenovo	63,014	**99.3%**	**51**	167	131	**1**	28,208	195	249,819	5.9
mockE	**SOAPdenovo_MA**	63,107	**99.3%**	**51**	**166**	131	**1**	28,208	195	249,819	**5.8**
mockE	Velvet	12,381	96.0%	41	269	106	2	46,122	128	183,815	9.2
mockE	**Velvet_MA**	12,830	96.2%	41	256	**100**	2	42,269	137	179,673	8.7
mockE	MetaVelvet	23,323	96.7%	49	474	160	5	62,131	93	**367,458**	13.0
mockE	**MetaVelvet_MA**	22,772	96.8%	49	462	156	4	**62,138**	**91**	**367,458**	12.7
mockE	Meta-IDBA	22,064	95.3%	47	362	151	3	26,141	223	249,069	11.0
mockE	**Meta-IDBA_MA**	22,032	95.4%	47	362	151	3	26,141	223	249,069	11.0
mockS	SOAPdenovo	45,251	**98.8%**	**28**	135	99	**0**	5,672	626	186,064	8.4
mockS	**SOAPdenovo_MA**	44,928	**98.8%**	**28**	135	98	**0**	5,672	626	186,064	**8.3**
mockS	Velvet	20,981	95.6%	**28**	498	127	1	6,134	770	119,120	22.4
mockS	**Velvet_MA**	21,050	95.8%	**28**	485	115	1	6,060	775	119,120	21.5
mockS	MetaVelvet	19,649	94.5%	**28**	518	158	2	13,028	**351**	**217,330**	24.2
mockS	**MetaVelvet_MA**	20,551	95.3%	**28**	517	143	3	6,685	622	**217,330**	20.1
mockS	Meta-IDBA	4,573	92.3%	18	**101**	**83**	0	**13,150**	368	119,604	10.2
mockS	**Meta-IDBA_MA**	4,559	92.5%	18	**101**	**83**	0	**13,150**	368	119,604	10.2
HMP	SOAPdenovo	39,028	**89.9%**	**11**	1,138	2,686	0	9,881	514	116,204	347.6
HMP	**SOAPdenovo_MA**	35,230	89.1%	**11**	1,138	2,618	0	**11,359**	**426**	**238,051**	**341.5**
HMP	Meta-IDBA	25,861	88.9%	7	718	2,102	0	4,215	1144	59,188	402.8
HMP	**Meta-IDBA_MA**	25,698	88.7%	7	**710**	**2,087**	0	4,215	1144	59,188	399.6
HMPscf	SOAPdenovo	31,673	99.9%	11	**-**	**-**	10	9,906	510	116,181	**0.9**
HMPscf	**SOAPdenovo_MA**	27,231	99.9%	11	**-**	**-**	10	11,359	426	**238,051**	**0.9**
HMPscf	Meta-IDBA	20,352	99.9%	**7**	**-**	**-**	10	4,946	939	59,188	1.4
HMPscf	**Meta-IDBA_MA**	22,886	99.9%	**7**	**-**	**-**	**9**	**22,304**	**238**	66,401	1.3

Datasets are mockE (mock Even), mockS (mock Staggered), HMP (Tongue dorsum, contig-level analysis), HMPscf (Tongue dorsum, scaffold-level analysis). All analyses other than HMPscf were done at the contig level. If necessary, contigs were extracted from scaffolds by splitting at three consecutive Ns. Assemblers with suffix _MA indicate the results produced by running MetAMOS on contigs produced by the corresponding assembler. #ctgs/scfs: total number of contigs/scaffolds in the assembly. Good Ctgs/scfs: fraction of contigs/scaffolds that mapped without errors to reference genomes. For the HMP dataset (Tongue dorsum contigs) alignments were only made to a small set of genomes estimated by the HMP project to match the genomes in this sample. For the HMPscf dataset good scaffolds are those without chimeric errors. Total Aln: total amount of sequence that can be aligned to the reference genomes (in Mbp). Slt: slight mis-assemblies determined by alignments that cover 80% or more of the aligned contig in a single match. Hvy: heavy misassemblies determined by alignments that cover less than 80% of the aligned contig in a single match or have two or more matches to a single reference. Ch: Chimeras are contigs with matches to two distinct reference genomes. Neither heavy mis-assemblies nor chimeras count towards reference coverage. Size @ 10 Mbp: the size of the largest contig *c *such that the sum of all contigs larger than *c *is more than 10 Mbp (similar to the commonly used N50 size). #@ 10 Mbp: smallest number of contigs whose cumulative size adds up to more than 10 Mbp. Max ctg size: size of the largest contig in the assembly. Err per Mbp: average number of errors per Mbp. Numbers in bold represent the best value for the specific dataset.

The results highlight the difficulty of choosing an appropriate assembler for a specific application. Depending on the dataset, different assemblers achieve the best trade-off between contiguity and errors. By allowing the reproducible execution of each assembler within a unified, automated framework, MetAMOS facilitates a more informed choice of assembler for any given application.

#### Assembly-based taxonomic annotation of reads

We evaluated the taxonomic annotations generated by FCP within MetAMOS on the mock communities using the same assemblers as above: SOAPdenovo, Velvet, MetaVelvet, and Meta-IDBA. We ran both the Annotate (taxonomic classification of contigs) and Propagate (classification propagation across scaffolds) steps with default parameters and compared results to the true read assignments determined by mapping reads to the known reference genomes with Bowtie [49,50]. The results shown in Table 2 demonstrate that performing the annotation after assembly within MetAMOS significantly reduces the number of unclassified reads and reduces the number of errors, irrespective of the assembly tool being used. Furthermore, the scaffold-based propagation of annotations further improves the results, leading to more reads being annotated while only slightly increasing the misclassification rate.

**Table 2:** Performance comparison of metagenomic annotation of reads versus contigs

			Class level (pre-propagate)			Class level (post-propagate)		
				
Dataset	Assembler	Run time (speedup)	Number unclassified	Number correctly classified	Number incorrectly classified	Number unclassified	Number correctly classified	Number incorrectly classified
mockE	None	84.2 h (-)	11,116,265	3,920,471	681,801	NA	NA	NA
mockE	**SOAPdenovo_MA**	33.0 h (2.6×)	634,091	14,852,561	231,885	612,517	14,874,157	231,863
mockE	Velvet_MA	29.4 h (2.9×)	870,073	14,611,333	237,130	854,554	14,626,870	237,112
mockE	**MetaVelvet_MA**	29.9 h (2.8×)	709,938	14,800,318	208,281	693,142	14,811,333	214,062
mockE	MetaIDBA_MA	37.8 h (2.2×)	1,700,699	13,652,114	365,724	1,676,319	13,676,524	365,724
mockS	None	167.1 h (-)	18,081,508	5,200,170	849,672	NA	NA	NA
mockS	**SOAPdenovo_MA**	72.3 h (2.3×)	1,971,900	21,772,125	387,325	1,850,541	21,884,121	386,688
mockS	Velvet_MA	71.8 h (2.3×)	2,392,898	21,313,998	424,454	2,250,852	21,456,487	424,011
mockS	**MetaVelvet_MA**	54.4 h (3.1×)	2,301,985	21,449,129	380,236	2,134,599	21,614,171	382,580
mockS	MetaIDBA_MA	53.8 h (3.1×)	2,576,941	21,316,513	237,896	2,210,972	21,681,036	239,342

Datasets are mockE (mock Even) and mockS (mock Staggered). Representing the truth, a total of 15,718,537/22,735,802 (69.14%) sequences could be unambiguously mapped using Bowtie for the mockE dataset and 24,131,350/39,918,454 (60.45%) for the mockS dataset. Assembler: each assembler was run within MetAMOS and the output contigs were classified using FCP. In the None case, the read sequences were classified by FCP prior to assembly. Classifications of reads with no known truth were neither penalized nor rewarded. Run time: **t**he time required to run either FCP on the reads or the Preprocessing, Assembly (for a specific assembler), Annotate and Propagate steps within MetAMOS is reported in CPU hours. The speedup factor is the FCP run time divided by the time required to perform the analysis within MetAMOS. All experiments were performed on a 64-bit Linux server equipped with eight 2.8 GHz dual-core processors and 128 GB RAM. Number unclassified, Number correctly classified, and Number incorrectly classified: total count of sequences, either unclassified, correctly classified, or incorrectly classified at the class taxonomic level. When compared to the unassembled results, classification within MetAMOS yields at least a three-fold increase in correctly classified sequences and a two-fold reduction in incorrectly classified sequences. Number unclassified, Number correctly classified, and Number incorrectly classified (post-propagate): the MetAMOS propagate step was used to transfer the annotations using the assembly graph. The total number of correctly classified sequences increases slightly in all cases, while not significantly increasing the number of incorrectly classified sequences. The full classification at each taxonomic level is given in Table S1 in Additional file 1. NA, not applicable.

### HMP tongue dorsum

#### Assembly of a tongue dorsum sample from the HMP project

Our second analysis was performed on real data (HMP tongue dorsum female sample, SRS077736). Velvet and MetaVelvet were not able to complete using 256 GB of memory, the maximum available to us; therefore, we restrict our results to SOAPdenovo and Meta-IDBA. For this sample we do not know the actual genomes comprising the community; instead we used the reference genome set identified by the HMP to have high similarity to the sequences within the sample (HMP Shotgun Community profiling SRS077736). This dataset was previously assembled with Meta-IDBA [25] and the published results demonstrated that Meta-IDBA was able to generate larger contigs than SOAPdenovo [51].

**Figure 5:**
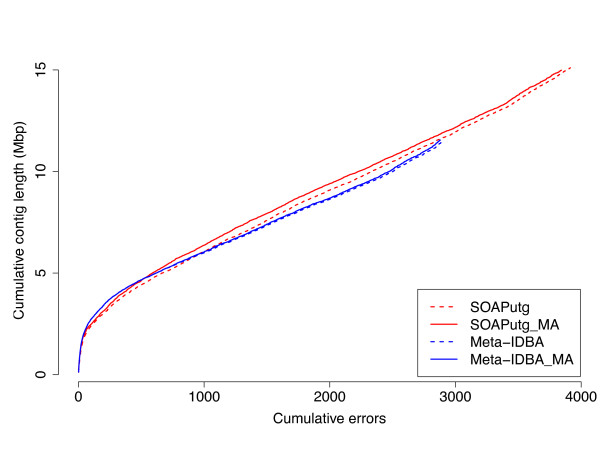
**Feature response curve for the HMP tongue dorsum sample**. The y-axis shows the cumulative contig length (sorted in decreasing order) and the x-axis shows cumulative errors. Curve includes all errors (slight, heavy, chimera; see text for more details).

To evaluate the correctness of these assemblies, we aligned them against the set of reference genomes and tabulated assembly errors. Unlike the mock datasets, the recruited references may not exactly match the true genomes in the sample. To allow for structural rearrangements within the same genome, we ignored errors occurring within the same reference genome (contigs with multiple alignments to the same reference) and only focused on chimeric errors (contigs spanning two or more reference genomes). Furthermore, we allowed higher rates of nucleotide errors in the alignment. None of the contigs were chimeric at the genus level or above. While both assemblers (SOAPdenovo and Meta-IDBA) vary in their ability to reconstruct individual genomes, MetAMOS is able to maintain or improve upon the starting assembly in all cases (Figure 5, Table 1).

**Figure 6:**
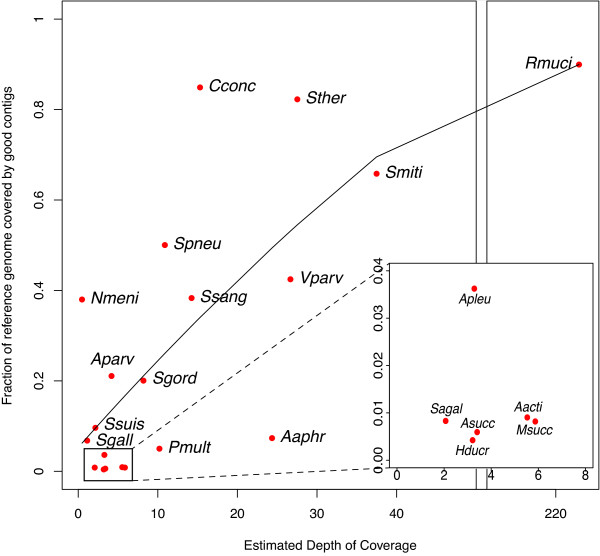
**Comparing depth of coverage versus percentage of reference covered by assembly on the HMP tongue dorsum sample**. The points represent individual reference genomes similar to the organisms in the sample. The x-axis represents the estimated depth of coverage while the y-axis represents the breadth of coverage (percentage of the reference covered by correctly assembled contigs). The coverage and percent-assembled values are significantly correlated (Spearman correlation coefficient 0.66, *P *= 0.002). A regression line is calculated using R scatter.smooth() function with a Gaussian model and span = 1.2. Genome names are abbreviated as follows: Aacti, *Aggregatibacter actinomycetemcomitans *D11S-*1*; Aaphr, *Aggregatibacter aphrophilus *NJ8700; Apleu, *Actinobacillus pleuropneumoniae *serovar 7 *str*. AP76; Aparv, *Atopobium parvulum *DSM 20469; Asucc, *Actinobacillus succinogenes *130Z; Cconc, *Campylobacter concisus *13826; Hducr, *Haemophilus ducreyi *35000HP; Msucc, *Mannheimia succiniciproducens *MBEL55E; Nmeni, *Neisseria meningitides *ATCC 13091; Pmult, *Pasteurella multocida subsp. multocida str*. Pm70; Rmuci, *Rothia mucilaginosa DY-18*; Sagal, *Streptrococcus agalactiae *18RS21; Sgall, *Streptococcus UCN34*; Sgord, *Streptococcus gordonii str. Challis substr. CH1*; Smiti, *Streptococcus mitis *B6; Ssang, *Streptococcus sanguinis *SK36; Ssuis, *Streptococcus suis *BM407; Spneu, *Streptococcus pneumonia *AP200; Sther, *Streptococcus thermophilus *LMD-9; Vparv, *Veillonella parvula *DSM 2008.

Using the tongue dorsum dataset we also explored the dependence between assembly quality and the relative abundance of an organism within a sample. As expected, the assembly quality strongly depends on the overall depth of coverage (Figure 6). Most reference genomes that were covered at < 5 to 10× were poorly assembled (reference coverage of 40% or less). These results hold irrespective of the assembler used (data not shown), indicating a fundamental limitation of assembly-based approaches for low abundance genomes. The abundance/coverage estimates obtained by mapping to reference genomes were consistent with those produced by MetAMOS using the taxonomic profiling tool MetaPhyler [52]. Thus, the taxonomic profiling data reported by MetAMOS provides a reference-independent means to assess which organisms in a sample can be effectively assembled.

The results described above are based on contig-level analyses in order to allow a comparison of multiple assemblers (for example, meta-IDBA does not perform scaffolding and would be unfairly penalized by a scaffold-level analysis). To demonstrate the value of the additional information contained in the scaffolds produced by MetAMOS we also performed an analysis of the contiguity and correctness of MetAMOS scaffolds compared to SOAPdenovo scaffolds and Meta-IDBA contigs (Table 1). As a complete collection of reference genomes is not available for this dataset, we only focused on chimeric errors - specifically contigs or scaffolds that map to two or more different reference genomes. When starting with either SOAPdenovo or Meta-IDBA contigs, MetAMOS was able to create more contiguous sequence, measuring a 200% improvement over the largest SOAPdenovo scaffold, 11% improvement over the largest Meta-IDBA contig, and over 5-fold increase in contiguity within the top 10 Mbp of the assembly, while making the same or fewer chimeric errors.

#### Biological variant identification

**Figure 7:**
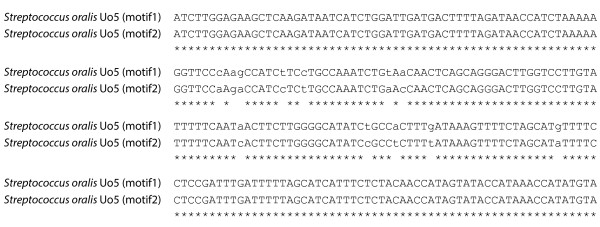
**HMP tongue dorsum variant motif**. This is a pairwise sequence alignment of a variant region between two closely related *Streptococcus oralis *strains. Matching alignment columns contain an asterisk underneath the column while columns with substitutions are indicated in lower case. The two motifs depicted in the image, motif1 and motif2, were automatically detected and output by MetAMOS.

MetAMOS, through its use of the Bambus 2 scaffolder [30], is currently the only metagenomic assembly pipeline able to automatically identify assembly patterns indicative of genomic variation (termed 'variation motifs'). Figure 7 shows an example section of a variant motif (spanning 1,212 bp) automatically reported by MetAMOS. This motif is composed of two variant sub regions, each 200 bp in length, connecting to two larger, 500 bp contigs in the assembly graph. Nucleotide alignments (using BLASTN, e-value < 10e-7) yield significant hits to *Streptococcus oralis *Uo5 and *Streptococcus sanguinis *SK36. The variant region in the middle contains 12 SNPs that fall within a poorly characterized hypothetical protein, distantly related to a glutamic acid decarboxylase (GAD) protein. GAD proteins (*gadB*) have been previously reported to show divergence in closely related strains of *Streptococcus thermophilus *[53]. This simple example highlights the utility of variation motifs. Typical assembly software would break the assembly in this region or forcefully merge the two variants into a mosaic (due to 'bubble popping' procedures). Instead, MetAMOS preserves the contiguity of the genome's backbone while also outputting the pattern of variation detected in this region. Note that such regions are difficult to identify within the output of existing assemblers, requiring substantial manual examination of the assembly output [54].

### Sexual dimorphism in the human gut microbiome

To demonstrate the types of analyses enabled by MetAMOS, we next investigate sexual dimorphism in the human gut microbiome. Microbiome differences between different genders were previously investigated in macaques [55] and mice [56], and such differences have yet (to the best of our knowledge) to be explored in humans. To explore whether evidence of sexual dimorphism could be gleaned from metagenomic data analyzed with MetAMOS, we focused on six subjects (three male and three female), all of the same age (59 years), and from the same country (Denmark), whose microbiome was sequenced as part of the MetaHIT project (sample details provided in Materials and methods). Note that conclusively assessing whether sexual dimorphism within gut bacteria of the human population requires extensive studies outside of the scope of this manuscript. Nevertheless, we decided to focus on this problem because: (a) to the best of our knowledge such an analysis has not been previously performed; and (b) the overall analysis approach is typical of a wide range of comparative metagenomic analyses that are commonly performed in a clinical setting.

The male and female samples, comprising more than 70 million sequences each, were analyzed with MetAMOS in under 4 days, using 20 cores and 128 GB of RAM. The maximum contig and scaffold sizes are similar, while the males have a slightly higher total number of assembled bases. MetaPhyler [52] was run both on the individual reads, pre-assembly, and on the final collection of ORFs, post-assembly. The taxonomic profiles pre-, and post-assembly are highly concordant (Spearman's correlation coefficient of 0.998 and 0.993 for the male and female samples, respectively). We estimate that MetaPhyler analysis on contigs requires roughly 300 times less computational resources than the equivalent analysis on the reads alone, highlighting the power of assembly as a data 'compression' tool, and suggesting that many analyses currently performed on the reads directly (for example, functional annotation [57,58], or pathway analysis [59]) could be substantially accelerated if performed on the assembled data instead.

#### Comparative analysis of multiple samples

**Figure 8:**
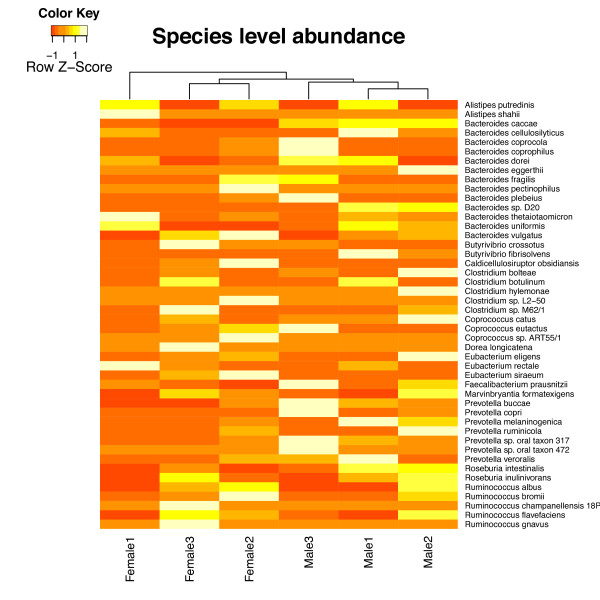
**MetAMOS comparative heat map for sexual dimorphism study**. Heat map calculated on the species-level diversity in the gut microbiome of six healthy Danish adults (as reported by MetaPhyler; full species listing for this experiment available for download from the MetAMOS website). Only taxa with abundance > 1% in all samples are reported. Sex is indicated on the x-axis, while individual species are labeled on the y-axis. The gradient from dark to light indicates the z-score of the abundance from low to high.

**Figure 9:**
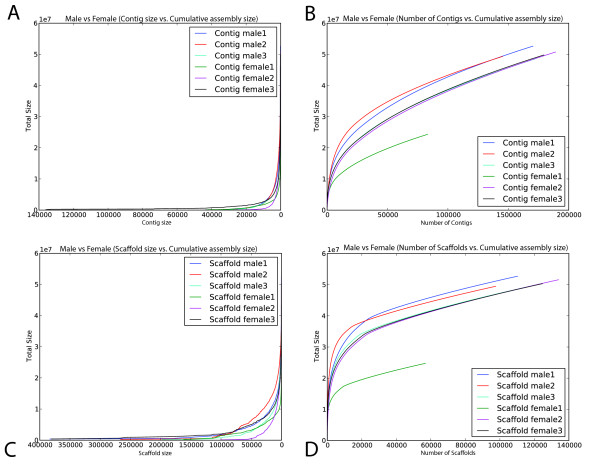
**MetAMOS comparative assembly report for sexual dimorphism study**. This plot display results from multiple female and male samples inside a single plot. The assembly comparison plots can be generated from multiple MetAMOS runs, allowing for comparisons of different samples or different assembly strategies. **(a) **Contig size versus cumulative assembly size. **(b) **Number of contigs versus cumulative assembly size. **(c) **Scaffold size versus cumulative assembly size. **(d) **Number of scaffolds versus cumulative assembly size.

MetAMOS includes utilities for performing comparative analyses of multiple assembled samples. To illustrate this functionality, we compare the taxonomic composition of the male and female samples in Figures 8 and 9, generated automatically within the MetAMOS HTML reports. Figure 8 contains a heat map of the taxonomic composition at the species level (calculated with MetaPhyler); Figure 9 shows assembly contiguity plots for contigs and scaffolds on multiple female and male assemblies. Our analysis reveals a higher predominance of members from the Bacteroidales order and a depletion of members from the Clostridiales order in male samples. This difference is not statistically significant at the order level; however, the family Eubacteriaceae and genus Eubacterium from the Clostridiales order are significantly depleted in males (*P *= 0.04 and *P *= 0.02, respectively, Fisher's exact test; *P *= 0.024 and *P *= 0.024, Metastats [60]. A statistical enrichment of *Bacteroides *can also be identified within the previously reported macaque data [55] (*P *= 0.0048, Metastats [60]). The comparative reports produced by MetAMOS also allow the visual comparison of the assembly statistics through accumulation plots (Figure 9).

#### Scaffold-based propagation of annotated reads

As briefly discussed earlier, MetAMOS includes a novel component responsible for propagating annotations to unclassified contigs. Using this procedure allowed us to assign taxonomic labels to an additional 985 contigs (from 918 to 1,903, a more than 2-fold increase) and to label 25 contigs as ambiguous on the female sample. Whenever the read-pair neighbors of a contig do not have a consistent annotation, MetAMOS marks the node as ambiguous, highlighting the conflicting annotation. Six of these contigs (all under 1 kbp in length) had received taxonomic labels when analyzed with the PhyloSift package and were re-classified as ambiguous by MetAMOS. We confirmed (using BLASTN and BLASTX searches with default parameters) that all of the contigs originate from ribosomal DNA sequence, supporting their ambiguous assignment as ribosomal repeats that can cause assemblers to incorrectly 'bridge' between unrelated organisms.

## Discussion

The goal of MetAMOS is to provide an integrated environment for metagenomic assembly and analysis, relying on both existing and novel algorithms and software tools. Results on both 'mock' communities with known sequence composition and real metagenomic data demonstrate that MetAMOS can generate accurate and contiguous assemblies of metagenomic datasets and improve the quality of initial assemblies constructed either with conservative assemblers developed for single genomes (for example, Velvet), or with assemblers specifically targeted at metagenomic data (for example, MetaVelvet).

Overall, our results indicate that the choice of assembler has a strong influence on the final assembly results and choosing the ideal assembler requires taking into account both contiguity and correctness. More aggressive assembly approaches sometimes result in more contiguous assemblies, but often introduce errors of the most severe kind (chimeras). The level of improvement provided by MetAMOS over other assembly tools is highly dependent on the specific characteristics of the dataset being assembled. MetAMOS only provided a small improvement over other tools (particularly SOAPdenovo and MetaIDBA) in the HMP mock communities; however, in the HMP tongue dorsum dataset the improvement was more pronounced. This result can be explained in part by the library size re-estimation automatically performed within the MetAMOS preprocessing stage (the library size reported in the NCBI Sequence Read Archive (SRA) was correct for the mock community but incorrect for the tongue dorsum), as well as by the ability of MetAMOS (through the use of Bambus 2) to effectively build scaffolds across regions of genomic variation. Such regions were substantially more abundant in the real dataset (approximately 10,000) compared to the artificial communities (approximately 300).

Thus, given a novel metagenomic dataset with unknown taxonomic composition, it can be difficult to choose an appropriate assembler *a priori*. This motivates our focus on fast end-to-end analysis and inclusion of multiple assembly methods, allowing the user to tailor the pipeline to their data. The modular design of MetAMOS enables its adaptation to new types of data by simply incorporating genome assembly tools tuned to the specific features of the new data.

In addition to assembly, MetAMOS provides several features important for downstream analysis, including taxonomic profiling, gene detection, and identification of genomic variation motifs. We have shown that the combination of these analyses within a single pipeline can produce improved results. We have demonstrated this for taxonomic classification, where analysis within MetAMOS increases the number of correctly classified sequences by as much as four-fold while reducing error. Another benefit is a two- to three-fold speed up within MetAMOS versus the taxonomic annotation methods run on the reads (Table 2). MetAMOS makes it straightforward to assess the performance of the various tools for each of these steps for a given sample. We continue to work on improving the performance of MetAMOS and plan to add additional analysis modules in future releases, including integration with pipelines for metabolic profiling (such as HUMAnN [61]).

Finally, the modular design and open-source licensing model enable researchers to adapt MetAMOS to new applications beyond our initial focus on metagenomic data. As an example, the combination of Velvet-SC (a single-cell assembler already integrated within MetAMOS) and the coverage-independent repeat detection methods of Bambus 2 make MetAMOS an effective pipeline for single-cell genomics. In addition to our primary goal of providing biologists with an integrated analysis pipeline for metagenomic data, we hope that the availability of MetAMOS will encourage researchers to contribute their own analysis modules, and that this framework will reduce duplication of efforts and accelerate developments in this field by allowing scientists to focus their attention on individual components without having to re-implement all the components of a metagenomic pipeline.

## MetAMOS computational design

MetAMOS was designed to be run in two modes: 'Assembly mode', which requires larger amounts of RAM and starts from raw read data; or 'Analysis mode', which starts from already assembled contigs/scaffolds and can be run on much more modest computational nodes or servers. MetAMOS has support for eight assemblers (SOAPdenovo [51], Newbler, Velvet [62,63], Velvet-SC [64], MetaVelvet [24], Meta-IDBA [25], CABOG [33] and Minimus [40]); six read/contig annotation methods (PhyloSift [65], BLAST [66], FCP [22,67], PHMMER [68], PhymmBL [14,15]); three metagenomic gene prediction tools (FragGeneScan [69], MetaGeneMark [35], Glimmer-MG [70]); one abundance estimation method (MetaPhyler [52]); a BLAST-based [66] functional annotation step using the Uniprot database [71]; a scaffolder engineered specifically for metagenomic data (Bambus 2 [30]); and an interactive tool for visualizing taxonomic and functional composition (Krona [46]) (Figure 1).

### MetAMOS workflow

Our design of the MetAMOS pipeline was motivated by two guiding principles: modularity and robustness. We intended to encourage users to tailor MetAMOS to the biological questions they want to answer, not the inverse. Given that each metagenome assembly/analysis presents a unique set of challenges/goals, users can take advantage of this modularity and customize their own pipelines by combining the modules they deem necessary. MetAMOS leverages a previously published workflow management system (Ruffus [29]) to track inputs/outputs/states and checkpoint while running through computationally intensive analyses. While MetAMOS offers several novel features specific to metagenomic assembly, we also wanted to leverage existing methods and software for metagenomic analysis to create a 'playground' for metagenomic assembly and encourage cooperation among the community. Upon download of the MetAMOS source and installation of python (2.5.x to 2.7.x), users only need run the *INSTALL *script. This will automatically configure the pipeline to run within the user's environment and also fetch all required data, if a connection to the Internet is available. Once installed, there are two main executables that comprise MetAMOS: *initPipeline *and *runPipeline*. *initPipeline *is mainly involved with creating a project environment, and describing input files (454/Illumina reads, assembled contigs) and library types. *runPipeline *takes a project directory as the input and will initiate execution of the entire MetAMOS pipeline (Figure 1). Next, we describe each step/module of the pipeline in detail.

#### Preprocess (required)

This is the starting point of all analyses in the pipeline. MetAMOS can take a variety of inputs, including interleaved and non-interleaved FastQ/FastA format, SFF files, and even a set of pre-assembled contigs. MetAMOS supports existing read-analysis tools such as FastQC [72] to evaluate the quality of the supplied read data. Preprocess includes an optional 'aggressive' read filter that discards any read containing 'N's or a base below a pre-defined quality value. The justification for aggressive read filtration is that read coverage/depth is no longer at a premium and the quality of reads has a huge influence on the quality of assemblies [73]. This initially may seem extreme, especially since this step can discard upwards of 25% of the reads; however, given the dependency of de Bruijn graph-based assemblers on clean data, we anticipate that assembly quality will be improved and have observed this in practice. We also include a read filtration step based on the fastx_toolkit [74] that allows the user to trim rather than discard the reads. Another important component of Preprocess is the library verification step that will check whether read pairs are properly aligned and also modify read headers to ensure they are compatible with downstream tools.

#### Assemble (optional)

Once reads are pre-processed they are passed to the Assemble step. Currently MetAMOS has support for eight assemblers, including SOAPdenovo, Newbler, Velvet, Velvet-SC, MetaVelvet, Meta-IDBA, CABOG and Minimus. Each of these assemblers has its own set of parameters and required input format, all of which are automatically managed within the pipeline and transparent to the user. It is our goal to keep growing this list to include the plethora of existing assemblers and eventually allow the user to combine assemblies via an assembly merging strategy, combining the strengths of each assembler and hopefully avoiding the weaknesses of any single strategy. Three types of assembly are possible in the current version of MetAMOS: (a) single genome/isolate, (b) metagenomic, or (c) single cell. While the main focus is on metagenomic assembly, thanks to the modular nature of MetAMOS, all three types are supported via the mentioned assemblers and command-line options. If pre-assembled contigs are input to MetAMOS, the assembly step is automatically disabled.

#### MapReads (required)

This step is necessary for MetAMOS. We currently rely on Bowtie [49] and Bowtie2 [50] for mapping all reads back to assembled contigs. This step is an essential step in the pipeline which performs the following tasks: (i) depth of coverage estimation; (ii) filtering of contigs with no reads mapping to them; (iii) creation of links for the scaffolding step; and (iv) re-estimation of fragment length for each provided paired-read library. This step is an important quality control step that helps to avoid propagating genome assembler mistakes downstream to later steps in the pipeline. Some assemblers do offer read positions that are directly usable for scaffolding downstream (CA, Velvet), and we preserve this information if requested by the user.

#### FindORFS (optional)

Following the MapReads step, we pass the contigs to the metagenomic gene prediction module. Three metagenomic gene prediction tools are currently supported: FragGeneScan, MetaGeneMark, and Glimmer-MG. The rationale for calling genes at this step is that most metagenomic gene prediction tools have significantly increased sensitivity and accuracy once the fragment is longer than 300 bp. Even though these tools are efficient, we limit the work by only calling ORFs on contigs with more than 3× depth of coverage and larger than 300 bp (both parameters are configurable by the user).

#### FindRepeats (optional)

A novel feature in MetAMOS, this step takes ORFs or contigs as an input and serves three purposes. First, it annotates repetitive contigs that can be used to identify under-collapsed sequence output by the assembler. Second, it allows for clustering predicted ORFs into families sharing high identity (> 97%), which may represent gene duplication events. Third, this step will allow us to bypass the MarkRepeat step of Bambus 2 (discussed below in further detail), which, depending on the sample, can become computationally expensive. Repetitive contigs are identified by the *de novo *repeat family detection algorithm implemented in Repeatoire [75]. Repeatoire relies on a probabilistic multi-alignment algorithm based on spaced seeds, which can handle indels and substitutions.

#### Annotate (optional)

This step takes ORFs or contigs as an input and determines which organisms comprise the given sample. In order to annotate the ORFs or contigs, we offer five classification methods spanning a range of techniques: homology-based (BLAST), composition-based (FCP), hidden Markov models (PHMMER, PhyloSift), and interpolated Markov models (PhymmBL). Annotating each and every read in a sample can be computationally prohibitive and lead to inaccurate classifications due to a lack of a discriminatory signal from such short sequences [21]. Thus, our philosophy is to assemble first and then classify contigs, or even better, ORFs. This allows a more focused approach to annotation that permits more reliable classification on the predicted ORFs compared to individual short reads. However, as not all reads find their way into the final assembly, any reads not mapped to the assembly in the MapReads step are labeled singleton reads. These reads are also classified in order to get a complete picture of the taxonomic composition of the sample.

#### FunctionalAnnotation (optional)

This step takes ORFs or contigs as input and determines what biological functions are present. To address this, we currently support homology-based (BLAST) functional assignment using the UniProt/Swiss-Prot database. Results from this step are displayed as a Krona chart of functional abundance in the HTML summary report. Note, however, that this step provides just a preliminary functional profile that should be refined using one of the many existing pipelines for this purpose (using the collection of ORFs output by MetAMOS in/FindORFS/out/proba.fna as an input). Accurate functional annotation is a complex task that is beyond the scope of our pipeline.

#### Scaffold (required)

The entanglement of repeats and genomic variation is one of the main challenges in metagenomic assembly. In clonal bacterial genome assembly, any regions that tangle the assembly graph are necessarily repetitive regions in the genome, and there exist a variety of strategies for disambiguating and resolving repeats in this context [76]. However, in metagenomic assembly, the tangles in the graph are not solely due to repeats and can also be caused by variable regions within closely related strains inside the community. Bambus 2 relies on a graph-based repeat detection method that can distinguish between repeat-induced tangles and likely regions of genomic variation without using prior knowledge of the taxonomic composition. Once repeats are identified and classified, Bambus 2 can focus on the variant regions in the assembly. Bambus 2 outputs these variant regions and makes them available to the user for downstream analysis.

#### Classify (optional)

One of the final steps of the MetAMOS pipeline assigns final classifications to each and every contig/ORF/scaffold in the outputs produced by earlier steps and stores them in subdirectories labeled at some pre-specified taxonomic level (class by default). In addition, all reads that were used in the assembly of the contigs/scaffolds are placed in each appropriate subdirectory. This step enables the easy identification of assembled contigs from taxonomic groups of interest, as well as the identification of DNA from potentially novel organisms.

#### Propagate (optional)

Another novel contribution of the MetAMOS pipeline is annotation propagation. We rely on the scaffold graph generated by Bambus 2 to transfer annotations (in our case taxonomic labels) to un-annotated contigs within the same scaffold. This process allows us to label contigs that cannot be reliably classified due to their short length. The assembled scaffolds are not modified during this step.

#### Postprocess (required)

Postprocess involves the generation of all reports and output into a single location (/Postprocess/out/). Once MetAMOS is finished running, if the user prefers to rerun any step with a different method, the pipeline can be re-run with the same command and the Ruffus [29] framework will ensure that only the necessary steps are run again. This allows for a quick exploratory run to be performed that is later refined once initial information is gathered on the composition and characteristics of the metagenomic community.

#### HTML summary report

The MetAMOS pipeline ends by generating an interactive, HTML summary (Figure 2) of the assembly statistics and estimated abundance information. The Summary page provides a graphical interface for navigating the data and reports generated by the pipeline. It comprises a main console surrounding a dynamic pane that can show specific reports for each step. These reports consist of tables, charts, and interactive Krona charts for exploring hierarchical abundance information. We offer several plots that allow for comparisons of different samples or different assembly strategies. Plots that are currently supported include: contig size versus cumulative assembly size, number of contigs versus cumulative assembly size, scaffold size versus cumulative assembly size, and number of scaffolds versus cumulative assembly size (Figure 9). As reports for each step are viewed in the dynamic pane, the main console persistently provides an overview of the run, including statuses of each step as well as summary statistics and charts. Details of the pipeline configuration and commands that were run are also accessible from the main console.

## Materials and methods

### Assembly validation

MUMmer [77] version 3.23 was used to align assembled contigs/scaffolds to the reference genomes (--maxmatch -l 20). When scaffolds were available, contigs were extracted by splitting the scaffolds at three or more consecutive Ns. For the scaffold analysis in the HMP tongue dorsum sample scaffolds were left intact. Only contigs/scaffolds over 150 bp were used for validation (unassembled reads did not count towards the total). Alignments were then filtered using 'delta-filter -i 97 -q' to only retain the best hits to the reference for each contig/scaffold. All statistics were calculated on the final set of filtered alignments using a custom validation script. A contig with an alignment to a single reference genome across its entire length (allowing for a ± 15 bp mismatch at the ends of the alignment) was considered a good contig. A contig with an alignment covering > 80% of the contig length but < 100% was considered a slight mis-assembly and still considered valid. A contig with single alignment covering less than < 80% of the contig length, multiple alignments to a single reference genome, or multiple alignments to multiple reference genomes were all considered as mis-assemblies (and in the case of alignments to multiple reference genomes, chimeric). For the HMP tongue dorsum dataset, contigs were allowed to have multiple alignments to a single reference and to align at lower identity (-i 90) due to the expected differences between the selected reference genome set and the actual genomes in the sample. None of the heavily mis-assembled contigs or chimeric contigs were used to calculate reference coverage statistics. The assembly validation scripts are available for download at [78]. For the scaffold analysis we only counted detectable chimera events as errors.

### Annotation validation

The mock dataset annotations were generated using FCP. Each assembler was run within MetAMOS as described below and the assembled contigs (along with unassembled sequences) annotated using FCP. To establish a truth, sequences were mapped using Bowtie to the known references. The command 'bowtie -p 10 -f -l 25 -e 140 --best -k 1 -S' was used to pick the best genome for each sequence. Unmapped sequences were also recorded. The annotation results were compared to this truth at each taxonomic level using custom scripts. Finally, the MetAMOS propagation step was run using the class-level annotation and the results compared to the pre-propagation results.

### Default MetAMOS parameters

The Preprocess step includes one external software program, FastQC, in addition to a custom filtration script for read pre-processing. By default, read pre-processing is disabled. If enabled (via the -t parameter), all reads containing low quality bases and Ns are aggressively discarded, which can result in 5 to 10% (or more) of the reads being discarded. If fastq files are available and FastQC is enabled (by default it is disabled), the following command is executed: fastqc -t < cpus > fastq_input_files. The assemble step currently supports eight assemblers. The default parameters/recipes for each assembler are available in the configuration files from the MetAMOS code repository and are listed below. The map reads step relies on the short read mapper bowtie to align the reads to the assembled contigs. The default bowtie command is: 'bowtie -l 25 -e 100 --best --strata -m 10 -k 1'. Alternatively, the user can select via the '-w' parameter to trim the reads to 25 bp and align with the following parameters: 'bowtie -v 1 -M 2'. Currently MetAMOS supports three metagenomic gene finders, MetaGeneMark, FragGeneScan, and Glimmer-MG. MetaGeneMark and FragGeneScan are run with default parameters. We rely on a utility script that runs Repeatoire to identify repetitive contigs and create multi-alignments of ORF families. Repeatoire parameters are, by default, set to: '--minreplen = 200 --z = 17'. By default, MetaPhyler is enabled to quickly estimate the abundance on the supplied metagenomic sample. The included version, MetaPhylerV1.13, relies on blastp. The blastp parameters used are: '-m 8 -b 10 -v 10'. No other parameters are required for running MetaPhyler. If annotate is enabled, FCP is used to annotate/classify contigs and predicted ORFs. The default parameters are used. In addition to FCP, we also support phmmer (-E 1.0e-10), PhyloSift ('all -threaded'), and PhymmBL (default program parameters). Bambus 2 is the metagenomic scaffolder included within MetAMOS and is also executed with default parameters (coverage cut-off is automatically calculated from the assembly graph).

### Software packages and corresponding parameters used in our experiments

#### Program versions

All parameters used were default unless otherwise specified. The parameters below are the defaults within the MetAMOS pipeline for each tool. Modifications to default program parameters were the result of either a) recommendations from the program's author/user guide, b) published parameter settings on similar datasets, or c) empirical studies. SOAPdenovo, version 1.05 was run with the parameters '-D -d -R -M 3'. Velvet version 1.1.05 was run with 'k = 51'. Meta-IDBA version 0.19 with parameters '--mink 21 --maxk < user specified > --cover 1 --connect'. MetaVelvet version 1.1.01 with default parameters. Bowtie version 0.12.7 was run with '-l 25 -e 100 --best --strata -m 10 -k 1'. MetaGeneMark version 2.7d was run with default parameters. FragGeneScan version 1.16 was run with default parameters. FCP (nb-classify, epsilon-NB.py) version 1.0 was run with default parameters.

#### HMP mock experiment

For all experiments, the default MetAMOS parameters were used. For all assemblers, a *k*-mer of 51 was specified. For Bambus 2, a redundancy threshold of 10 was used.

#### HMP tongue dorsum experiment

For all experiments, the default MetAMOS parameters were used (except for specifying alternative assemblers with -a soap for SOAPdenovo and -a metaidba for Meta-IDBA). For all assemblers, a *k-*mer of 51 was specified. For Bambus 2, a redundancy threshold of 10 was used. The motif was aligned using web-based blastn against the nr database to identify top-scoring genes.

#### MetaHIT experiment/sexual dimorphism

We selected three males and three females randomly from the MetaHit project having the same age (59 years), the same country (Denmark), and the same enterotype (ET1) [7]. We also chose the samples to have approximately equal body mass index (26.19 for males versus 24.12 for females). The chosen samples were MH0041, MH0045, and MH0055 for males and MH0002, MH0024, and MH0082 for females. MetAMOS was run on all three samples of each sex using the longer paired libraries for each sample (ERR011181, ERR011189, ERR011209 for males and ERR011091, ERR011149, ERR011264 for females).

To test for concordance between pre- and post-assembly annotations, we selected the order level classifications and compared the percentage classified at each order in the pre- and post-assembly male and female samples independently. We used R (version 2.11.1) and the command cor.test(preAsm, postAsm) to estimate the concordance between pre- and post-assembly assignments. To test for significance of the difference between samples we used the Fisher exact test on the order, family, and genus compositions of the male and female samples with the R command fisher.test(x).

We ran two versions of MetaPhyler, one based on BLAST in addition to the new version based on MUMmer. The new MetaPhyler is significantly faster; the new MetaPhyler ran in 12 CPU hours compared to 25 CPU hours for the post-assembly analysis (which used the original MetaPhyler).

For all experiments, the default MetAMOS parameters were used and a *k*-mer of 51 was specified. For Bambus 2, a redundancy threshold of 10 was used.

### Datasets used in our experiments

HMP mock samples were part of the Human Microbiome Project Metagenomes Mock Pilot (BioProject ID: 48475) and available for download at [79].

HMP tongue dorsum sample was downloaded from the SRA [SRA:SRS077736].

MetaHIT human gut metagenome samples: *MH0041 [80] (run accession ERR011181) [81,82]; *MH0045 [83] (run accession ERR011189) [84,85]; *MH0055 [86] (run accession ERR011129) [87,88]. MetaHIT human gut metagenome samples from three Danish females (aged 59 years): *MH0002 [89] (run accession ERR011091) [90,91]; *MH0024 [92] (run accession ERR011149) [93,94]; *MH0082 [95] (run accession ERR011264) [96,97].

## Availability

MetAMOS is available from [78]. MetAMOS and AMOS-specific code are released open source under the Perl Artistic License [98]. Licensing restrictions for bundled software are outlined on the main project page. Operating systems are Mac OS X and most UNIX systems and programming languages are Perl, Python, Java, C++.

## Abbreviations

bp: base pair; GB: Gigabyte; HMP: Human Microbiome Project; MetaHIT: Metagenomics of the Human Intestinal Tract; HTML: Hyper-Text Markup Language; kbp: thousand base pairs; Mbp: million base pairs; ORF: open reading frame; SNP single-nucleotide polymorphism; SRA: Sequence Read Archive.

## Competing interests

The authors declare that they have no competing interests.

## Authors' contributions

MP, AP, SK, and TT conceived the project and designed the experiments and software. BO and AD contributed to the design of experiments and software. TT, SK, DS, BO, and AD implemented and tested the software. TT, SK, IA, BL, and AD performed the experiments. SK and TT collected and analyzed the results. MP, TT, SK and AP wrote the manuscript. All authors read and approved the final manuscript. SK and TT contributed equally to this work.

## Supplementary Material

Additional file 1 **Figure S1 and Table S1**. Click here for file
