# Supplementary material for: MetAMOS: a modular and open source metagenomic assembly and analysis pipeline
Source: Genome Biol. 2013 Jan 15;14(1):R2. doi: 10.1186/gb-2013-14-1-r2 (PMC4053804; doi:10.1186/gb-2013-14-1-r2)
Supplement: Additional file 1 — Figure S1 and Table S1. [file gb-2013-14-1-r2-S1.DOC]

Supplementary Figure S1. Feature response curve performance on mock Even (a) and mock Staggered (b) communities. The y-axis shows the cumulative contig length (sorted in decreasing order) and the x-axis shows the corresponding number of misassembled contigs. The thick lines (assemblies ending in _MA) represent the results obtained by running metAMOS using the corresponding assembler (dashed lines) for the Assembly module. A. HMP Mock Even, MetaVelvet assembler, all errors. B. HMP Mock Staggered, MetaVelvet assembler, all errors. C. HMP Mock Even, Velvet assembler, all errors reported. D. HMP Mock Staggered, Velvet assembler, all errors.

**Supplementary Table S1. Taxonomic-classification for the MockE and MockS samples for three assemblers.** **#Assembler** – Each assembler was run within MetAMOS, and the output contigs and singleton reads that could not be mapped to the assembly were also classified using FCP. In the None case, the sequences were classified by FCP. **# Unclassified, # Correctly Classified, and # Incorrectly Classified** – total count of sequences either unclassified, correctly, classified, or incorrectly classified at each taxonomic level. **Taxonomic Level** – the level of the taxonomy at which the classification accuracy is measured. **Total** – the total number of sequences with a true assignment.
